# Supplementary material for: Functional module detection through integration of single-cell RNA sequencing data with protein–protein interaction networks
Source: BMC Genomics. 2020 Nov 2;21:756. doi: 10.1186/s12864-020-07144-2 (PMC7607865; doi:10.1186/s12864-020-07144-2)
Supplement: Supplementary file 1 — Additional file 1 Supplementary Information. [file 12864_2020_7144_MOESM1_ESM.pdf]

Functional module detection through integration of single-cell RNA sequencing data with protein–protein interaction networks – Supplementary Information

Florian Klimm*[1], Enrique M. Toledo[2], Thomas Monfeuga[2], Fang Zhang[2], Charlotte M. Deane[1], and Gesine Reinert[1]

[1]Department of Statistics, University of Oxford, Oxford OX1 3LB, United Kingdom
[2]Discovery Technology and Genomics, Novo Nordisk Research Centre Oxford, Oxford OX3 7FZ, United Kingdom

March 6, 2020

1 Supplementary Note: Scoring Function

As in [1], we consider p-values $x \in [0,1]$ to follow a *beta–uniform mixture* (BUM) distribution which is a mixture of noise and signal component [2]. The noise follows a uniform distribution $U([0,1])$ with the *probability density function* (pdf)

$$f_U(x) = \begin{cases} 1\,, & \text{for } x \in [0,1] \\ 0\,, & \text{else}\,. \end{cases} \tag{1}$$

and the signal follows a beta distribution $B(\alpha, \beta = 1)$ with the pdf

$$f_B(x) = \begin{cases} \alpha x^{\alpha-1}\,, & \text{for } x \in [0,1] \\ 0\,, & \text{else}\,. \end{cases} \tag{2}$$

*Corresponding author. Email: f.klimm@gmail.com

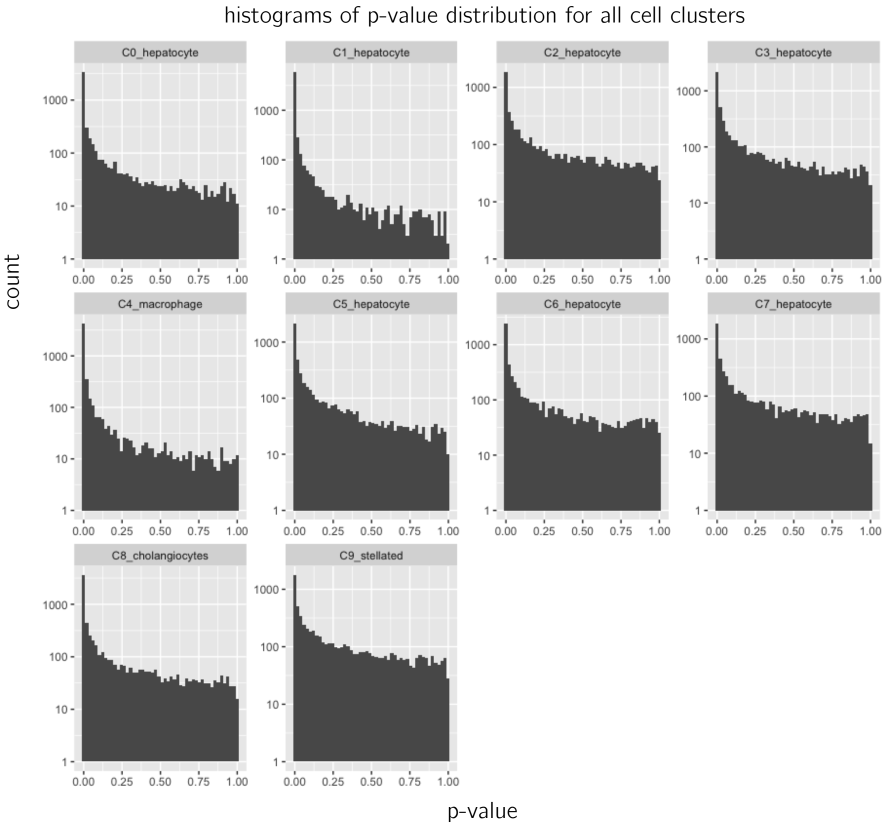

Figure 1: The distribution of p-values of differential expression for each cell cluster. As expected, they follow roughly a beta–uniform distribution.

The *shape parameter* $\alpha \in [0, 1]$ is a free parameter. We use $\lambda \in [0, 1]$ as *mixture parameter* to construct the distribution of p-values

$$f_{BUM}(x) = \lambda f_U(x) + (1 - \lambda) f_B(x) \tag{3}$$

$$= \begin{cases} \lambda + (1 - \lambda)\alpha x^{\alpha - 1}\,, & \text{for } x \in [0, 1] \\ 0\,, & \text{else}\,. \end{cases} \tag{4}$$

For each run of our algorithm, we obtain estimates of shape parameter α and mixture parameter λ by performing a maximum-likelihood estimation. For this, we use an iterative limited-memory Broyden-Fletcher-Goldfarb-Shanno algorithm [3].

Fig. 2 shows a histogram of the p-values for the one-vs-one comparison between clusters H1 and H3 and the fitted distribution. Fitting a BUM model yields a mixture parameter of $\lambda \approx 0.549$ and a shape parameter of $\alpha \approx 0.482$.

The BUM is in decent agreement with the observed p-values (a one-sample Kolmogorov–Smirnov test yields a statistic of $D \approx 0.014$ and a p-value of 0.086). The p-values for the other clusters follow roughly a beta–uniform distribution, too (see Fig. 1). We calculate the value of the pdf at $x = 1$, which represents the background noise, as

$$\tilde{f} = f_{BUM}(x = 1) = \lambda + (1 - \lambda)\alpha \approx 0.77\,, \tag{5}$$

and show is a dotted horizontal line in Fig. 2.

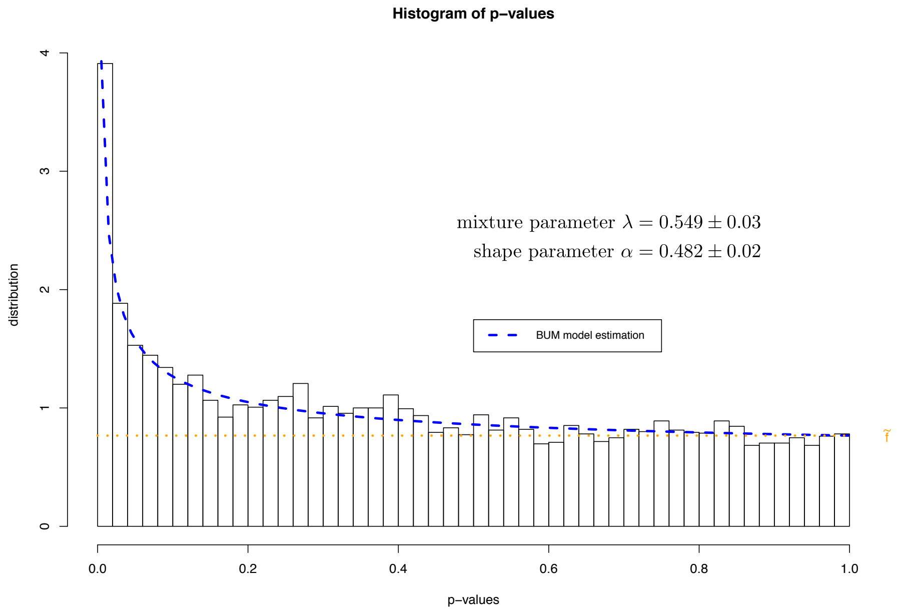

Figure 2: Fit of the BUM model to p-values for comparison cluster H1 vs cluster H3. We show a histogram of the observed p-values and the fitted BUM model (blue dashed curve). The mixture parameter is $\lambda \approx 0.549$ and the shape parameter of the beta distribution is $\alpha \approx 0.482$. We show the probability $\tilde{f} = f(x = 1) \approx 0.77$ as a horizontal dotted orange line.

We use an approach as outlined in [1] to construct an additive score $S(x) \in \mathbb{R}$ with negative values indicating background noise and positive values representing signal. This allows us to compute the differential expression of a set of genes by adding their associated scores. An appropriate choice is

$$S(x) = (\alpha - 1)\left(\log(x) - \log(\tau)\right)\,, \tag{6}$$

where we consider p-values below the *significance threshold* $\tau > 0$ as noise and those above as signal. As shown in [2], the significance threshold is a function of the FDR and we may estimate it for a BUM as

$$\tau(\text{FDR}) = \left(\frac{\tilde{f} - \text{FDR} \cdot \lambda}{\text{FDR}(1-\lambda)} \right)^{1/(\text{FDR}-1)} \quad \text{with } \tilde{f} = f_{BUM}(x=1) = \lambda + (1-\lambda)\alpha\,. \tag{7}$$

To summarise, the procedure for computation of the node scores from p-values is as follows:

1. estimation of mixture parameter λ and shape parameter α from the distribution of p-values,
2. choice of an FDR as appropriate for data of choice,
3. computation of the the appropriate significance threshold through Eqn. (7), and
4. computation of the node scores through Eqn. (6).

2 Supplementary Note: Optimisation

As outlined in Supplementary Note 1, we assign each gene a score $S \in \mathbb{R}$. Under the null hypothesis, these scores are independent and identically distributed random variables. For any set Q of genes, we can compute the score

$$S_Q = \sum_{i \in Q} S(x_i)\,, \tag{8}$$

which indicates to what extent this set Q is significantly differently expressed.

The aim of our optimisation algorithm is the identification of a set of genes that maximises this score (i.e., being strongly differently expressed in a module) while the associated proteins are connected to each other in the PPIN. Mathematically, this problem is known as a *maximum-weight connected subgraph* (MWCS) problem.

Problem. (Maximum-weight connected subgraph, MWCS) *Given a vertex-weighted graph $G = (V, E, W)$, find a connected subgraph $T = (V_T, E_T, W) \subset G$ that maximises the score $W(T) = \sum_{i \in V_T} W(i)$.*

Here, we use $T \subset G$ to indicate that $T = (V_T, E_T)$ is a subgraph of G, i.e., $V_T \subset V$ and $E_T \subset E$. A graph is connected if there is a path between every pair of edges [4].

Finding a MWCS is NP-hard. A heuristic approach was used in [5] to identify the MWCS. It is computationally advantageous to transform this problem into an equivalent *prize-collecting Steiner tree* (PCST) problem because it is often possible to obtain provably optimal solutions for this transformed instance [1, 6].

Problem. (Prize-collecting Steiner tree, PCST) *Given a graph* $G = (V, E, c, p)$ *in which the edge weights* $c : E \to \mathbb{R}^{\geq 0}$ *indicate costs and the node weights* $p : V \to \mathbb{R}^{\geq 0}$ *indicate profits, find a connected subgraph* $T \subset G$ *that maximises the profit*

$$p(T) = \sum_{i \in V_T} p(v) - \sum_{e \in E_T} c(e)\,.$$

We can identify a MWCS by transforming the node-weighted network $G = (V, E, W)$ into a network $G = (V, E, c, p)$ with non-negative node and edge weights. Specifically, we compute $p(i) = w(i) - w_{\min}$ and $c(e) = -w_{\min}$, where $w_{\min} = \min_{i \in V(G)} w_i$, so that all edges have the same weight. See Fig. 3, for an example and [1] for a proof of this equivalence.

Here, we use the dual ascent-based branch-and-bound framework DAPC-STP [7, 8] to find the PCST. While this algorithm is not guaranteed to find an optimal solution, in practice it finds solutions close to optimality for networks with hundreds of thousands of nodes and millions of edges in minutes to hours. For all computations on the PPINs in this manuscript, we found optimal solutions in less than ten seconds.

PCSTs are always trees (i.e., if they have N nodes they have $N - 1$ edges) because adding additional edges to the PCST decreases the profit P. Accordingly, MWCSs are also trees. When visualising the active modules, however, we construct a *node-induced subgraph*, which is the set of nodes in T with all edges from G. Therefore the modules may contain loops.

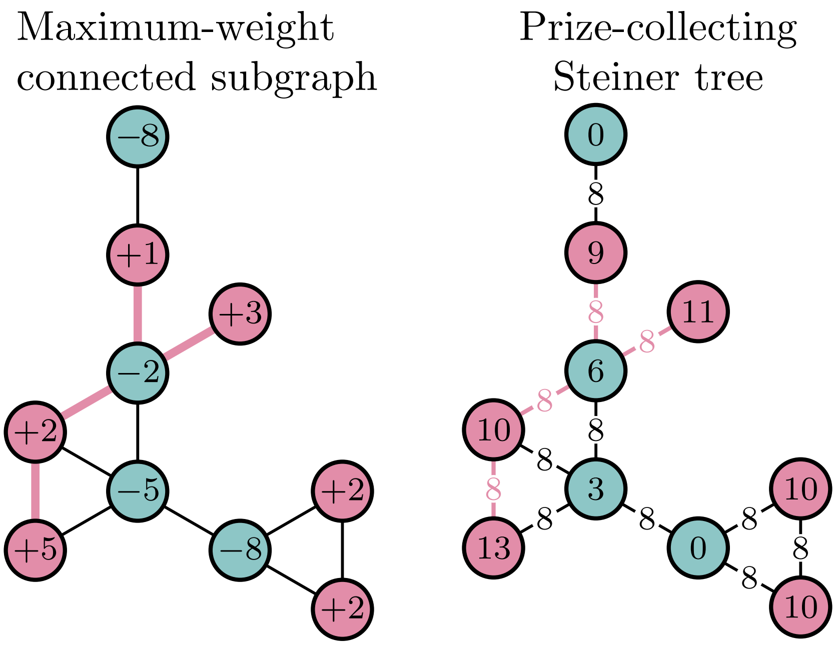

Figure 3: Example of a node-weighted network for which we want to find the MWCS (left) and its equivalent node- and edge-weighted network for which we find the PCST (right). We transform this problem by shifting all node weights by $w_{\min} = -8$ and assigning each edge a cost of $c = -w_{\min} = 8$. We show nodes with a positive score in the node-weighted network in red and nodes with negative score in blue. We highlight the optimal solution in both instances by colouring the edges red. The MWCS has a weight of $W = 5{+}2{-}2{+}3{+}1 = 9$ and the PCST has a profit of $P = 13 + 10 + 6 + 11 + 9 - 4 \times 8 = 49 - 32 = 17 = 9 + 8$.

2.1 Pairwise comparison between cell clusters

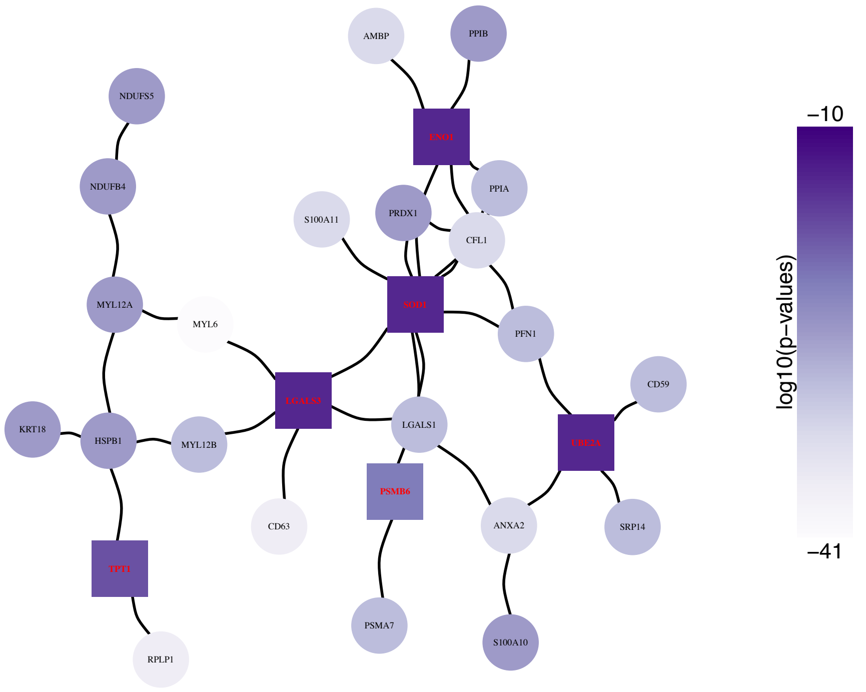

Figure 4: We can compare also the expression between two clusters. Here, we show the active module in cluster H2 vs cluster H4.

In the main manuscript, we have identified active modules in cell clusters by comparing the expression of genes against the expression of all other cells (‘one-vs-all’). It is also possible to compare the expression of any two clusters pairwise (’one-vs-one’) and obtain a potentially more nuanced picture of two clusters that have a similar but distinct transcriptional state.

In Fig. 4, we show the detected functional module in a pairwise comparison of clusters H2 and H4. To compute the p-values of differential expression between two clusters, we can also use the FindMarkers function in Seurat. The rest of the analysis pipeline in scPPIN is the same. We identify a module that consists of proteins such as TPT1, a tumour protein, and SOD1, which is involved in apoptosis.

We compare cluster H1 vs H3 and detect a functional module as shown in Fig. 5. Note that the FDR$=0.01$ is very large (in comparison with earlier calculations and FDR$=10^{-19}$ in the main manuscript) as both clusters have a very similar gene expression. A GO-term enrichment analysis finds only the term ’regulation of symbiosis, encompassing mutualism through parasitism’ enriched. Our method identifies TRIM25, an E3 ubiquitin ligase enzyme [9]. TRIM25

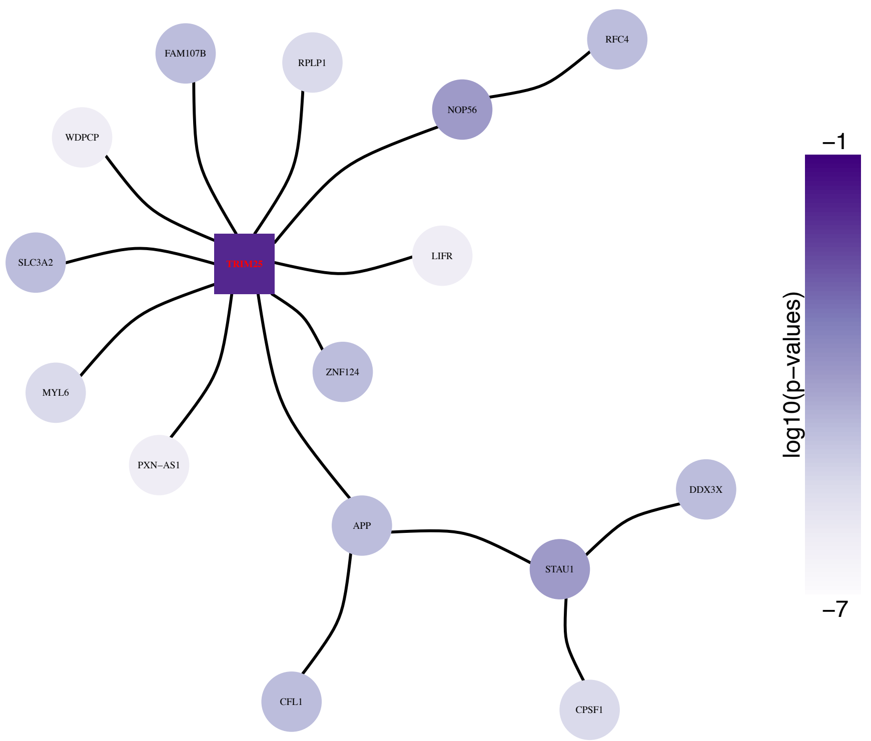

Figure 5: We can compare also the expression between two clusters. Here, we show the active module in cluster H1 vs cluster H3.

binds to viral RNA and so regulates immune response against viruses. These results indicate that either: one of the clusters is infected with a virus or this clusters shows a response to inflammation that is using pathways that are usually associated with antiviral response.

3 Supplementary Note: Missing expression data

In the method described in the main manuscript and Supplementary Note 1, we assign each node in a PPIN a score based on the p-value of differential expression. This is only possible if we have the gene-expression measurement for a given protein. In practice, we delete nodes that represent proteins without available expression value. One alternative approach is to assign each node in a PPIN for which we have no expression information of the corresponding gene, a small negative score (i.e., $S = S_0 = -1$). There is no *a priori* correct way to choose S_0 and smaller value decrease the likelihood that such proteins are part of the detected functional module. For $S_0 \to -\infty$, we recover the case without missing-data replacement.

In Fig. 6, we show a detected functional module for a node-weighted PPIN in which we assigned proteins without gene expression data $S_0 = -1$. It is the same underlying data and FDR = 0.01 as in Fig. 5. As before, we detect TRIM25 and interacting proteins. In addition, we also detect three proteins without expression information, RPA2, RBM4, and SOX2. The total number of proteins in this active module increases from 16 to 20. Note that for some of these additional proteins (e.g., JUND and RPP40), we have gene expression data but they were not in the MWST. We now detect them as part of the functional module because they are connected to the original module via the missing-data nodes.

This example demonstrates that incorporating nodes with no associated scRNA-seq data may alter the detected modules. The choice of S_0 is a free parameter and a principled approach for detecting them will be part of future research.

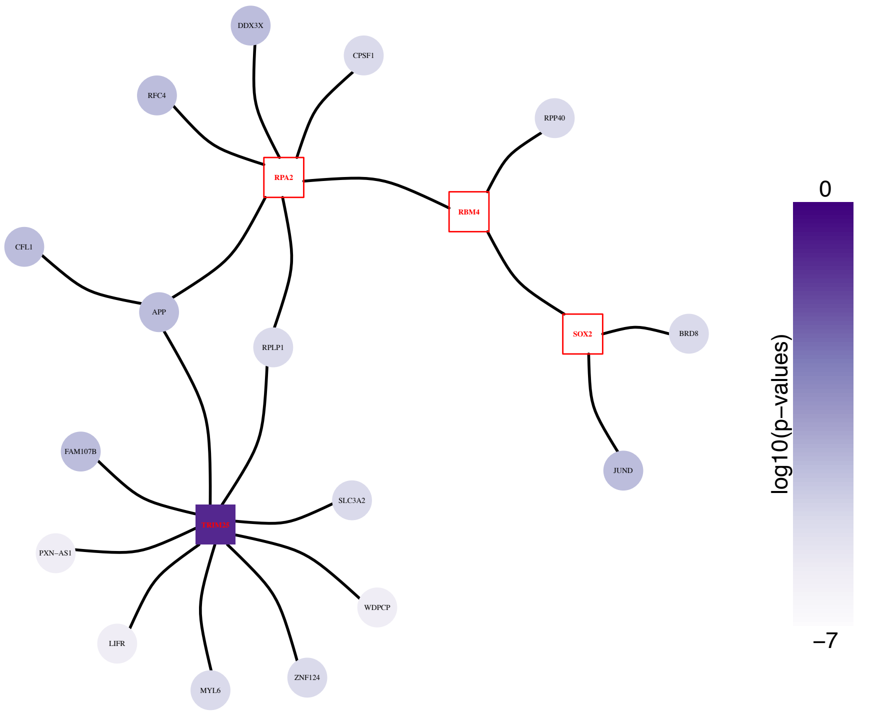

Figure 6: Assigning proteins without expression data for corresponding genes a score S_0 allows to keep these proteins in the node-weighted PPIN. Here, we show the active module in cluster H1 vs cluster H3 for $S_0 = -1$. In comparison with Fig. 5, we detect here three proteins without expression information: RPA2, RBM4, and SOX2 (shown as red boxes).

4 Supplementary Figure: Detected modules for six hepatocyte clusters

Fig. 7 shows the detected modules for all six hepatocyte clusters FDR $= 10^{27}$. It is identical to Fig. 4 in the main manuscript but with all proteins labelled.

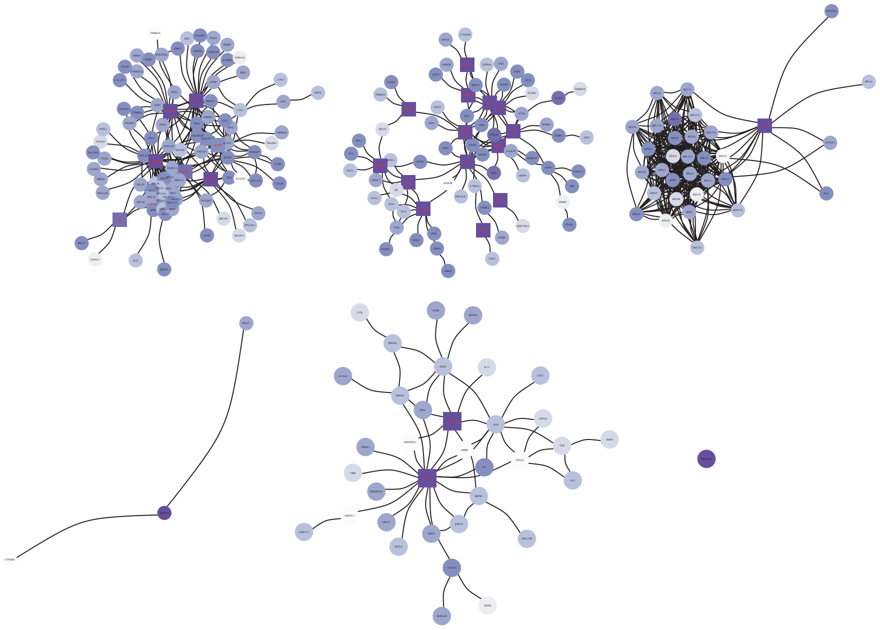

Figure 7: Detected modules for all six hepatocyte clusters for FDR $= 10^{27}$. We find that the detected modules vary strongly in size with the smallest consisting of a single protein and the largest consisting of 51 proteins. Colour indicates p-value of associated gene from low (white) to high (purple). We show nodes as squares if the could not have been detected without PPIN information.

5 Supplementary Note: Influence of FDR on size of detected modules

In the main manuscript, we show the influence of the FDR on the size of the detected module for one cluster. Here, we show this for all six clusters. For all clusters we compute the functional modules for FDR $\in [10^{-45}, 10^{-15}]$. Note that the horizontal axes are scaled differently for each cluster for illustration purposes.

For all clusters, we find that the size of the functional modules is increasing with the FDR. Furthermore, for all clusters, there are FDR-choices for which

we detect modules that have some proteins that we would not have detected from a DEG analysis alone.

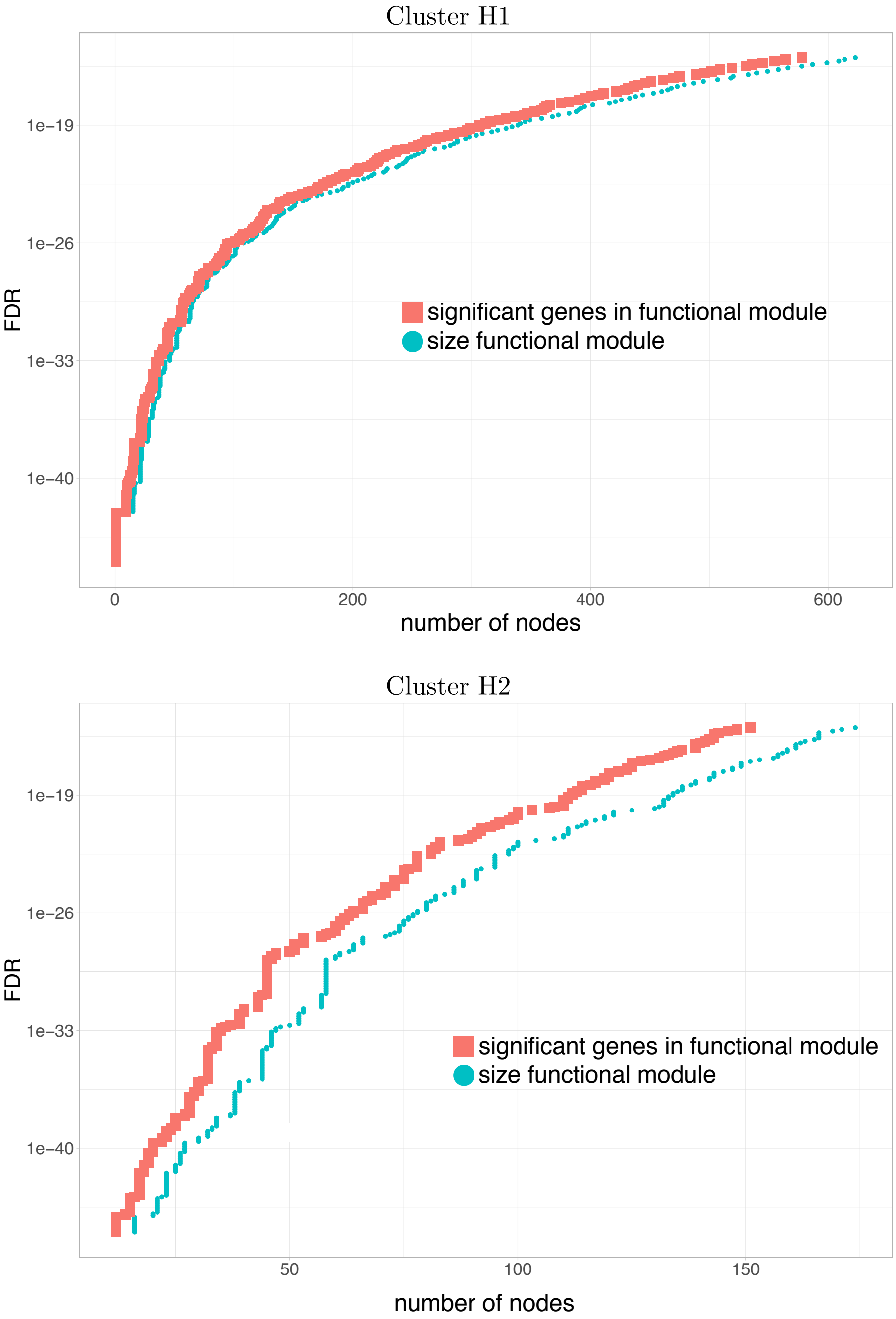

Figure 8: The module size as a function of the FDR for clusters H1 and H2.

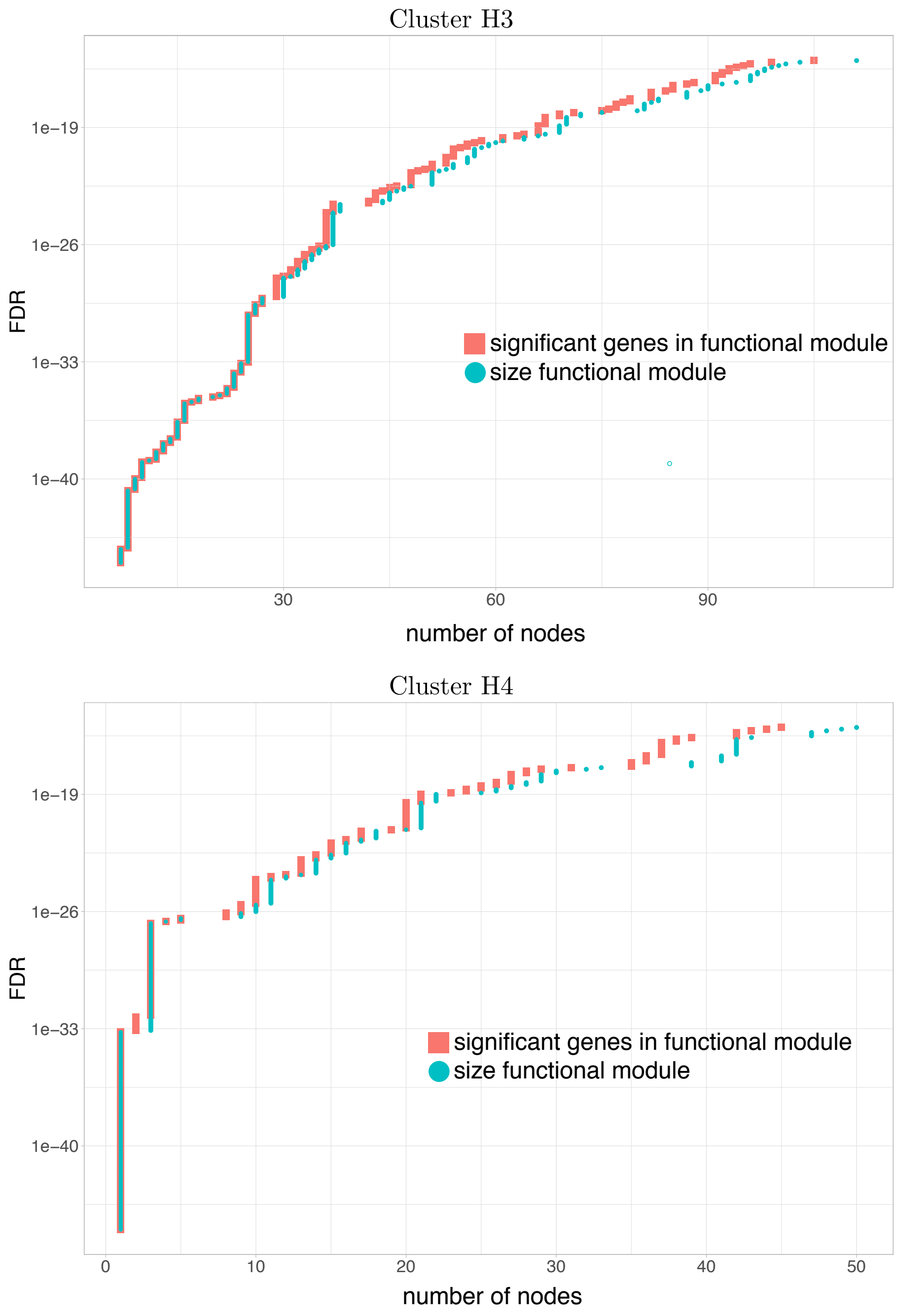

Figure 9: The module size as a function of the FDR for clusters H3 and H4.

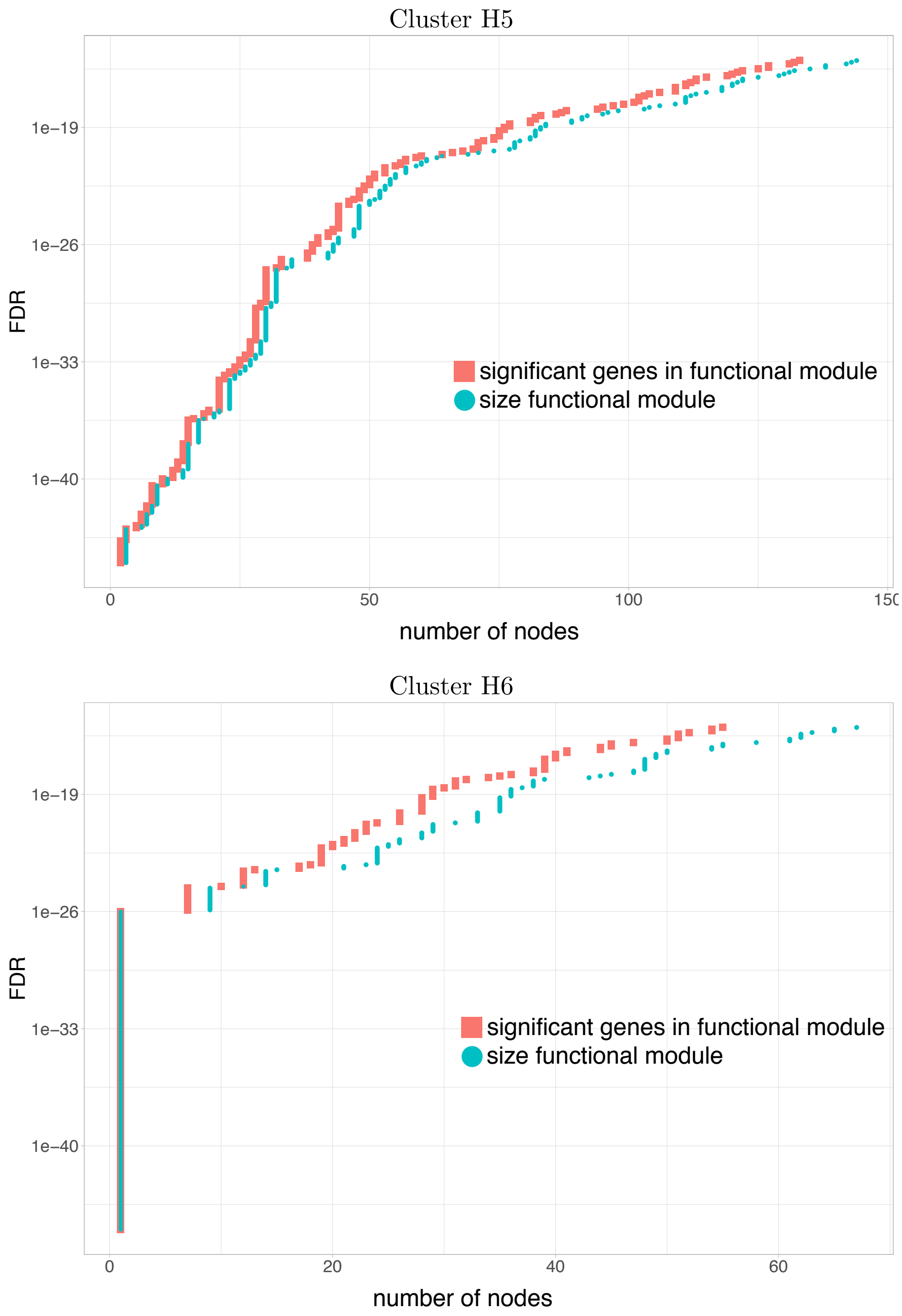

Figure 10: The module size as a function of the FDR for clusters H5 and H6.

6 Supplementary Results: Human adipose tissue

6.1 Data

Pre-processed single cell RNA-sequencing data from human adipose tissue was downloaded from GEO accession GSE129363 [10]. Only cells from non-diabetic patients were retained for analysis. The data was then processed similarly to the original publication. After clustering, clusters expressing high levels of CFD were kept and re-clustered using highly variable genes in this sub-dataset (3563 cells). Differential expression analysis for each cluster was performed as described in the main manuscript.

6.2 Functional modules

A modularity-based clustering (see Method section in main manuscript) with Seurat reveals four clusters. For each cluster, we compute a functional module with scPPIN and choose a FDR of 10^{-20}.

We show the detected modules in Fig. 11. The modules vary in size from 30 to 56. To test their biological significance, we perform a GO-term enrichment analysis (see Method section). We find that all of them have biological functions enriched (see Tab. 1). These reflect the distinct function of cells in the different clusters. Clusters 3 and 4, for example, are associated with the extracellular matrix organisation, which has been identified as one component of the tissue development [11]. Cluster 4 shows a specific response to wounding, which indicates a state-change as a result of a stimulus indicating damage to the organism [12]. Cluster 1 seems to be involved in generic signalling pathways as a response to a stimulus, while Cluster 2 is specific to cytokine signalling, which are a major regulator of adipose tissue metabolism [13].

In addition to testing the association of a module with biological function, we also compare it to disease annotations with Enrichr [14]. We find that that the module of cluster 3 is most strongly associated with 'familial partial lipodystrophy' ($p < 0.008$) and 'unspecified disorder of lipoid metabolism' ($p < 0.01$), both hinting at malfunction in the synthesis and degradation of lipids in the adipose cells. Somewhat surprisingly, Cluster 2 is associated with 'postmenopausal osteoporosis'. There exists, however, emerging evidence for an interaction between adipose tissue and the skeleton, which especially includes the secretion of cytokines [15]. This indicates that scPPIN is not only able to detect functional modules but also sets of genes that are associated with diseases.

7 R library scPPIN

The R library is available under `https://github.com/floklimm/scPPIN`. It contains multiple tutorials that describe the usage of the available functions.

cluster	module size M	top enriched GO terms	$\log_{10}(p\text{-value})$
1	43	response to organonitrogen compound	-8
		negative regulation of biological process	-7
		cell surface receptor signaling pathway	-7
		aging	-6
2	30	response to oxygen-containing compound	-8
		response to cytokine	-5
		regulation of cell migration	-4
3	50	extracellular matrix organization	-7
		protein targeting to ER	-7
		cell adhesion	-4
4	56	extracellular matrix organization	-7
		response to wounding	-5
		response to endogenous stimulus	-4

Table 1: For each of the four clusters we give, the size M, the most enriched GO terms and the multiple-testing corrected p-value.

The function DETECTFUNCTIONALMODULE(PPIN,PVALUES,FDR) can be used to directly compute the functional module in a PPIN. It has three input parameters: PPIN, which allows users to use a protein–protein interaction network of their choice; PVALUES, which is the list of p-values; and FDR, which is the false-discovery rate. We provide PPINs constructed from BIOGRID for sixty-eight organisms.

We also provide a tutorial on how to use SCPPIN in combination SCANPY [16].

References

[1] Marcus T Dittrich, Gunnar W Klau, Andreas Rosenwald, Thomas Dandekar, and Tobias Müller. Identifying functional modules in protein–protein interaction networks: an integrated exact approach. *Bioinformatics*, 24(13):i223–i231, 2008.

[2] Stan Pounds and Stephan W Morris. Estimating the occurrence of false positives and false negatives in microarray studies by approximating and partitioning the empirical distribution of p-values. *Bioinformatics*, 19(10):1236–1242, 2003.

[3] Dong C Liu and Jorge Nocedal. On the limited memory bfgs method for large scale optimization. *Mathematical Programming*, 45(1-3):503–528, 1989.

[4] Geir Agnarsson and Raymond Greenlaw. *Graph Theory: Modeling, Applications, and Algorithms.* Prentice-Hall, Inc., 2006.

[5] Trey Ideker, Owen Ozier, Benno Schwikowski, and Andrew F Siegel. Discovering regulatory and signalling circuits in molecular interaction networks. *Bioinformatics*, 18(suppl_1):S233–S240, 2002.

[6] Ivana Ljubić, René Weiskircher, Ulrich Pferschy, Gunnar W Klau, Petra Mutzel, and Matteo Fischetti. An algorithmic framework for the exact solution of the prize-collecting steiner tree problem. *Mathematical Programming*, 105(2-3):427–449, 2006.

[7] Matteo Fischetti, Markus Leitner, Ivana Ljubić, Martin Luipersbeck, Michele Monaci, Max Resch, Domenico Salvagnin, and Markus Sinnl. Thinning out steiner trees: a node-based model for uniform edge costs. *Mathematical Programming Computation*, 9(2):203–229, 2017.

[8] Markus Leitner, Ivana Ljubić, Martin Luipersbeck, and Markus Sinnl. A dual ascent-based branch-and-bound framework for the prize-collecting Steiner tree and related problems. *INFORMS Journal on Computing*, 30(2):402–420, 2018.

[9] María Martín-Vicente, Luz M Medrano, Salvador Resino, Adolfo García-Sastre, and Isidoro Martínez. TRIM25 in the regulation of the antiviral innate immunity. *Frontiers in Immunology*, 8:1187, 2017.

[10] Jinchu Vijay, Marie-Frédérique Gauthier, Rebecca L Biswell, Daniel A Louiselle, Jeffrey J Johnston, Warren A Cheung, Bradley Belden, Albena Pramatarova, Laurent Biertho, Margaret Gibson, et al. Single-cell analysis of human adipose tissue identifies depot-and disease-specific cell types. *Nature Metabolism*, 2(1):97–109, 2020.

[11] Ikuyo Nakajima, Hisashi Aso, Takahiro Yamaguchi, and Kyouhei Ozutsumi. Adipose tissue extracellular matrix: newly organized by adipocytes during differentiation. *Differentiation*, 63(4):193–200, 1998.

[12] Anna Franz, Will Wood, and Paul Martin. Fat body cells are motile and actively migrate to wounds to drive repair and prevent infection. *Developmental Cell*, 44(4):460–470, 2018.

[13] Simon W Coppack. Pro-inflammatory cytokines and adipose tissue. *Proceedings of the Nutrition Society*, 60(3):349–356, 2001.

[14] Edward Y Chen, Christopher M Tan, Yan Kou, Qiaonan Duan, Zichen Wang, Gabriela Vaz Meirelles, Neil R Clark, and Avi Ma'ayan. Enrichr: interactive and collaborative html5 gene list enrichment analysis tool. *BMC Bioinformatics*, 14(1):128, 2013.

[15] Masanobu Kawai, Francisco JA de Paula, and Clifford J Rosen. New insights into osteoporosis: the bone–fat connection. *Journal of internal medicine*, 272(4):317–329, 2012.

[16] F Alexander Wolf, Philipp Angerer, and Fabian J Theis. Scanpy: large-scale single-cell gene expression data analysis. *Genome biology*, 19(1):15, 2018.

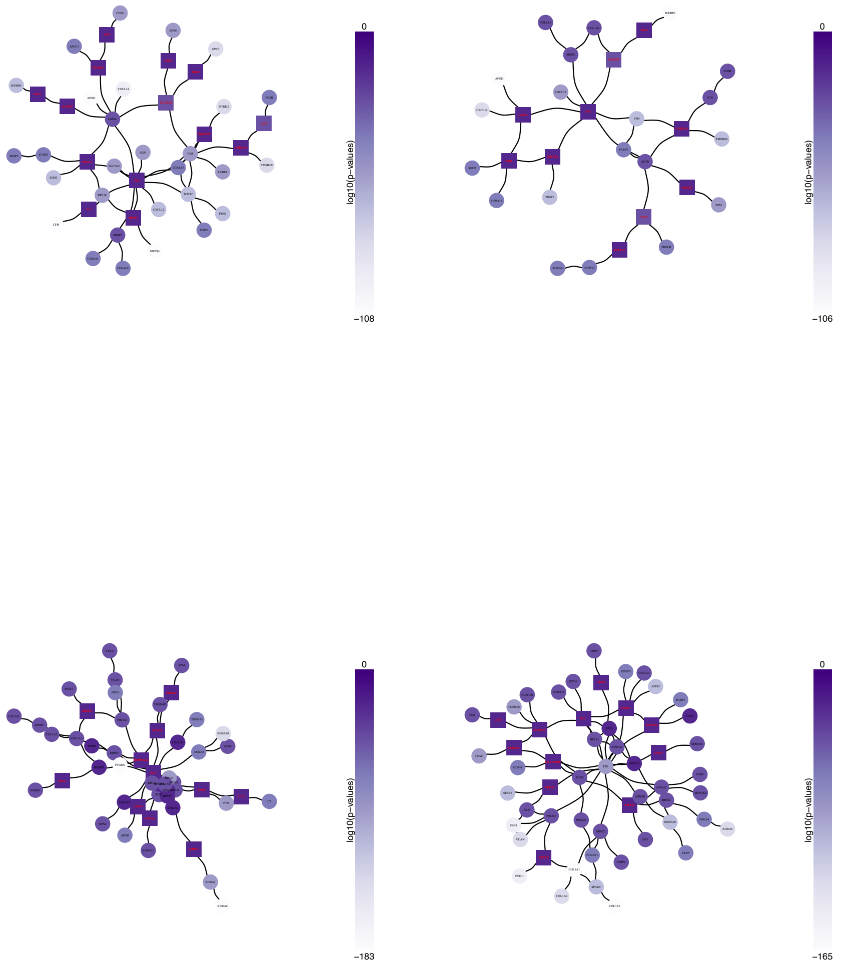

Figure 11: Functional modules in the four cell clusters in the adipose tissue data.
